# Regional Frontal Gray Matter Volume Associated with Executive Function Capacity as a Risk Factor for Vehicle Crashes in Normal Aging Adults

**DOI:** 10.1371/journal.pone.0045920

**Published:** 2012-09-19

**Authors:** Hiroyuki Sakai, Miwa Takahara, Naomi F. Honjo, Shun'ichi Doi, Norihiro Sadato, Yuji Uchiyama

**Affiliations:** 1 Information & Electronics Research Division, Toyota Central Research and Development Laboratories, Inc., Nagakute, Aichi, Japan; 2 Department of Human Informatics, Aichi Shukutoku University, Nagakute, Aichi, Japan; 3 Department of Radiology, Osaka Neurosurgical Hospital, Takamatsu, Kagawa, Japan; 4 Division of Intelligent Mechanical Systems Engineering, Kagawa University, Takamatsu, Kagawa, Japan; 5 Division of Cerebral Integration, National Institute for Physiological Sciences, Okazaki, Aichi, Japan; Banner Alzheimer's Institute, United States of America

## Abstract

Although low executive functioning is a risk factor for vehicle crashes among elderly drivers, the neural basis of individual differences in this cognitive ability remains largely unknown. Here we aimed to examine regional frontal gray matter volume associated with executive functioning in normal aging individuals, using voxel-based morphometry (VBM). To this end, 39 community-dwelling elderly volunteers who drove a car on a daily basis participated in structural magnetic resonance imaging, and completed two questionnaires concerning executive functioning and risky driving tendencies in daily living. Consequently, we found that participants with low executive function capacity were prone to risky driving. Furthermore, VBM analysis revealed that lower executive function capacity was associated with smaller gray matter volume in the supplementary motor area (SMA). Thus, the current data suggest that SMA volume is a reliable predictor of individual differences in executive function capacity as a risk factor for vehicle crashes among elderly persons. The implication of our results is that regional frontal gray matter volume might underlie the variation in driving tendencies among elderly drivers. Therefore, detailed driving behavior assessments might be able to detect early neurodegenerative changes in the frontal lobe in normal aging adults.

## Introduction

In general, physical and cognitive capabilities decrease with age among elderly individuals, and these age-related declines in functioning can be risk factors for vehicle crashes in elderly drivers (see [1] for review). However, this does not imply that every elderly individual over a certain age poses a threat while driving. Safety considerations for elderly drivers need to address individual differences in functional capabilities.

Driving is a complex process involving planning and execution of appropriate actions based on the comprehension of the traffic environment. Therefore, executive function is considered a primary cognitive ability for safe driving. In fact, Daigneault et al. [2] demonstrated that elderly drivers with a history of multiple accidents in the preceding five years displayed poor performance on cognitive tasks requiring executive function, compared with an accident-free control group. In addition, another group reported that performance on a specific visual task (useful field of view, UFOV), examining an executive aspect of visual attention, was capable of discriminating elderly drivers with a history of at-fault accidents in the preceding five years from accident-free elderly drivers [3]–[7]. Thus, individual differences in executive functioning are associated with vehicle crash risk, particularly among elderly drivers.

Brain morphometry studies have revealed relationships between individual differences in cognitive abilities and morphological variation in certain brain regions (see [8] for review). In extreme cases, such as in Alzheimer's disease, pathological memory decline accompanies severe brain atrophy, especially in the hippocampus [9]–[15]. Also, for executive functioning, Westlye et al. [16] found associations between task performance for controlling visual attention and cortical thickness of the anterior cingulate cortex (ACC), the inferior frontal gyrus and the dorsolateral prefrontal cortex in healthy young adults. Furthermore, van Gaal et al. [17] demonstrated that higher conflict solving performance, which is an executive function, is associated with a larger gray matter volume in the pre-supplementary motor area. Thus, variation in regional gray matter volume in these frontal control regions may underlie individual differences in executive functioning. However, currently available evidence for associations between frontal brain structure and executive function capacity in normal aging individuals is limited, and even controversial. Van Petten et al. [18] did not find any correlation between frontal gray matter volume and executive function capacity. In contrast, Duarte et al. [19] found a significant, albeit negative, correlation between left middle frontal gyrus volume and executive functioning. More recently, Elderkin-Thompson et al. [20] demonstrated that better executive functioning is associated with larger ACC volume, consistent with results obtained using healthy young adults [16], [17].

In this study, we aimed to examine regional frontal gray matter volume associated with executive functioning in normal aging individuals, using voxel-based morphometry (VBM). Executive functioning was evaluated using the Effortful Control Scale (ECS), which is a questionnaire used to quantify executive function capacity of daily living [21], [22]. In addition, driving behavior was assessed using the Driving Behavior Questionnaire (DBQ) [23], [24] to examine whether executive function capacity, assessed using the ECS, was associated with vehicle crash risk. Furthermore, regional gray matter volume in the frontal lobe was examined for correlations with the ECS score.

## Materials and Methods

### Participants

This study was undertaken as part of a research project toward the comprehensive understanding of risk factors for vehicle crashes in elderly drivers in Kagawa prefecture. A total of 48 community-dwelling elderly individuals participated in this project for financial compensation. All participants gave their written informed consent, and the institutional ethics committee of Toyota Central Research and Development Laboratories, Inc. approved the study protocol.

Among these, we selected 39 normal aging elderly participants (23 males and 16 females), aged 65–76 (69±3) years, based on the following neurological screening criteria: 24 or more on the Mini Mental Statement Examination and within normal limits for atrophy, ventricular dilation and white matter hyperintensities. The screening process was carefully conducted by an experienced neuroradiologist (one of the authors, NFH) in a formalized brain check-up system termed *Brain Dock*.

### Questionnaires

Executive function capacity was assessed using the ECS, which was originally a subscale of the Adult Temperament Questionnaire [22]. The ECS consists of 35 self-reported items (e.g., “It's often hard for me to alternate between two different tasks”) that are rated on a four-point scale (1: very false; 2: somewhat false; 3: somewhat true; and 4: very true). The total score for the 35 items was normalized by dividing by the total possible score of 140 points (for simplicity) and was used as an index of executive function capacity. Because participants in the present study were all Japanese, we employed the Japanese-translated version of the ECS, which has been demonstrated to have adequate internal reliability and high test-retest reliability [25].

Risky driving tendencies were assessed using the DBQ [23], [24] to determine if executive function capacity assessed with the ECS was associated with vehicle crash risk. The DBQ, which consists of 50 self-reported items on daily driving behaviors that are rated on a 6-point scale (1: never; 2: hardly ever; 3: occasionally; 4: quite often; 5: frequently; and 6: nearly all the time), can extract three types of aberrant driving behavioral tendencies. The first type, *violations*, corresponds to deliberate contraventions of traffic laws (e.g., “Disregard the speed limits late at night or early in the morning”). The second type, *errors*, corresponds to misjudgments and failures that can be hazardous to other road users (e.g., “Fail to notice that pedestrians are crossing when turning into a side street from a main road”). The third type, *lapses*, also corresponds to erroneous behaviors, but poses no threat to others (e.g., “Get into the wrong lane approaching a roundabout or a junction”). We employed the Japanese-translated version of the DBQ [26] and computed scores for violations, errors and lapses by simply summing relevant items. Each score was normalized to have a maximum value of 1, and a correlation coefficient with the ECS score was determined and statistically tested with a one-tailed Pearson's correlation test for each score. The significance level was set at 0.017 (0.05/3) after the Bonferroni correction for multiple comparisons.

### Voxel-based morphometry

High-resolution isotropic three-dimensional T1-weighted gradient-echo images of the brain were acquired using a magnetic resonance imaging (MRI) scanner (1.5-T Philips Intera Achieva; Philips Medical Systems, Best, Netherlands) with repetition time  = 12 ms, echo time  = 2.4 ms, flip angle  = 8°, field of view  = 256×256 mm, 160 contiguous sagittal slices, and spatial resolution  = 1×1×1 mm^3^.

The anatomical brain images were processed using the VBM8 toolbox (r435; http://dbm.neuro.uni-jena.de/vbm/), which was incorporated in the SPM8 software (http://www.fil.ion.ucl.ac.uk/spm/). The VBM8 involves bias correction, tissue classification and spatial normalization with diffeomorphic anatomical registration through exponentiated Lie algebra [27]. We used the default parameters of the VBM8. However, as an exception, the standard International Consortium for Brain Mapping space template for East Asian brains was used for affine regularization. As a result, segmented, normalized and modulated gray matter images were provided for subsequent VBM statistical analysis. Note that the modulation process was performed using the Jacobian determinants of the nonlinear deformations used for normalization in order that the voxel intensities reflected regional gray matter volumes adjusted for individual brain sizes. Finally, the modulated gray matter images were smoothed with an 8 mm full-width at half-maximum isotropic Gaussian kernel.

After preprocessing of anatomical brain images, gray matter volume in the frontal lobe was examined for potential correlations with executive function capacity. A mask image for the frontal lobe was created using the WFU PickAtlas toolbox (http://www.fmri.wfubmc.edu) incorporated in SPM8. Age and gender were considered as covariates of no interest to regress out their contributions to gray matter volume. The statistical criteria were set to *P*<0.005 at voxel level, with *P*<0.05 corrected for multiple comparisons using family-wise error at cluster level. Moreover, for each of the three risky driving tendencies assessed with the DBQ, VBM statistical analysis was performed using identical procedures and statistical criteria.

## Results

**Figure 1:**
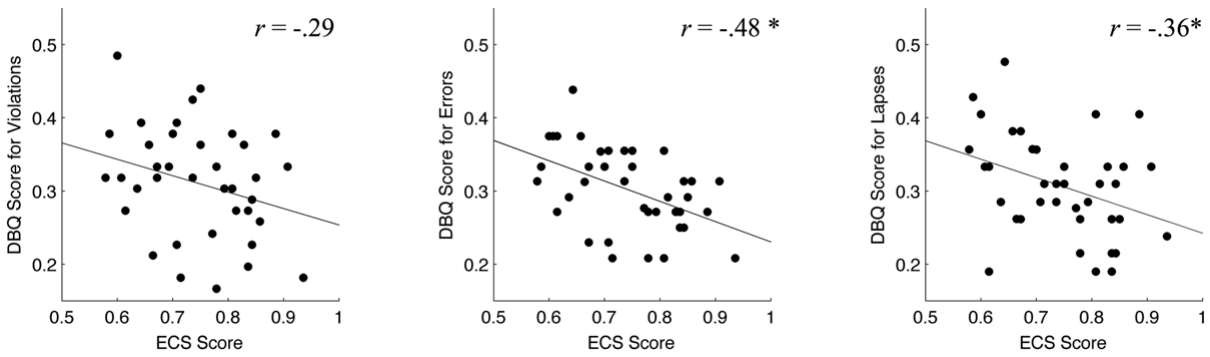
Scatter diagrams of Effortful Control Scale (ECS) score and Driving Behavior Questionnaire (DBQ) scores. Panels A, B and C denote DBQ scores for violations, errors and lapses, respectively, during daily driving. Each dot represents each participant. Asterisks indicate statistically significant correlations (*P*<0.05/3; one-tailed Pearson's correlation test adjusted by the Bonferroni correction for multiple comparisons).

The ECS scores showed modest significant negative correlations with DBQ scores for *errors* (*r* = −0.48, *P*<0.001) and *lapses* (*r* = −0.36, *P*<0.013), and a weak (not significant) negative correlation with a DBQ score for *violations* (*r* = −0.29, *P* = 0.035) across participants (Figure 1). These negative correlations between ECS and DBQ scores indicate that lower executive functioning is associated with greater risky driving tendencies in elderly drivers.

**Figure 2:**
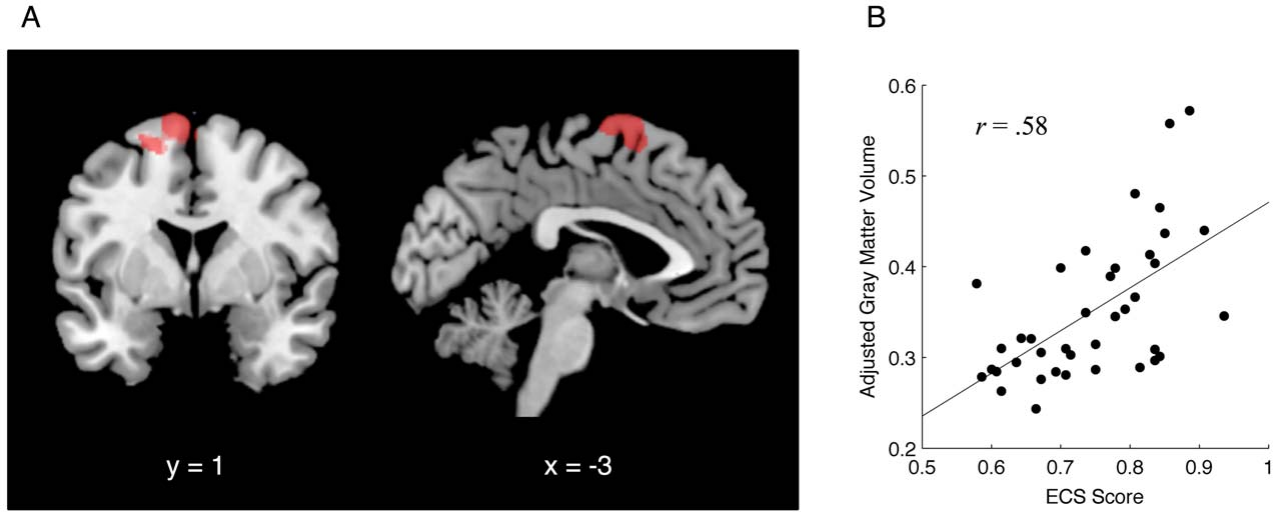
Brain area for which gray matter volume positively correlated with executive function capacity assessed with the Effortful Control Scale (ECS). The significant cluster (A) was identified based on statistical criteria of *P*<0.005 at voxel level, with *P*<0.05 corrected for multiple comparisons at cluster level. At the peak locus (MNI coordinates: *x* = −3, *y* = 1, *z* = 70; BA 6, supplementary motor area), the correlation coefficient between ECS score and gray matter volume, adjusted for age and gender, was 0.58 (B).

VBM analysis revealed that gray matter volumes in the supplementary motor area (SMA) were positively correlated with ECS scores (Figure 2A). At the peak locus (Montreal Neurological Institute coordinates: *x* = −3, *y* = −1, *z* = 70; BA 6), a correlation coefficient between ECS score and gray matter volume, adjusted for age and gender, was 0.58 (*P*<0.001, Pearson's correlation test; Figure 2B). The positive correlation indicates that lower executive function capacity is associated with smaller SMA volume.

**Figure 3:**
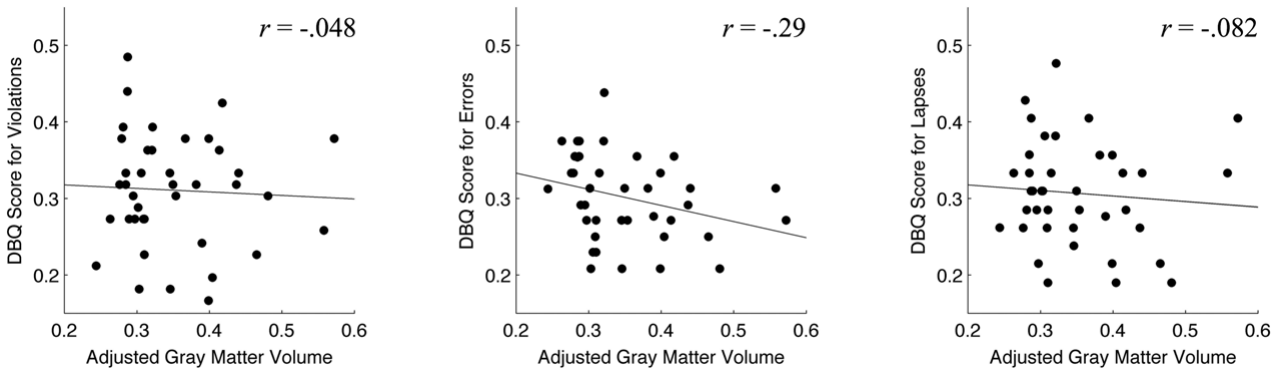
Scatter diagrams of Driving Behavior Questionnaire (DBQ) scores and gray matter volume at the peak locus (MNI coordinates: *x* = −3, *y* = 1, *z* = 70; BA 6, supplementary motor area) found in VBM analysis for executive function capacity. Panels A, B and C denote DBQ scores for violations, errors and lapses, respectively, during daily driving. Each dot represents each participant. No significant correlation was found (*P*>0.05/3; one-tailed Pearson's correlation test adjusted by the Bonferroni correction for multiple comparison).

In VBM analysis using DBQ scores, despite the fact that DBQ scores had a significant negative correlation with ECS scores, no cluster passed the statistical threshold corrected for multiple comparisons. Even at the peak locus extracted in VBM analysis using ECS scores, no significant correlations were found after the Bonferroni correction for multiple comparisons between gray matter volume and DBQ scores, although there was a weak negative correlation for a DBQ score for *errors* (*r* = −0.29, *P* = 0.035; Figure 3).

## Discussion

In the present study, we examined regional frontal gray matter volume associated with executive functioning in elderly drivers. We found a significant positive correlation between executive function capacity, assessed with the ECS, and gray matter volume in the SMA. In addition, we found that participants with a low ECS score were prone to risky driving. Thus, the current data suggest that SMA volume is a reliable predictor of individual differences in executive function capacity as a risk factor for vehicle crashes in the elderly.

### Behavioral data

We adopted the ECS to assess executive function capacity. In many previous studies, however, executive function capacity has been measured using cognitive task performance. For instance, the Stroop task [28] and the Eriksen flanker task [29] are used to assess the conflict solving aspect of executive function; whereas the Attention Network Task [30] and the UFOV task [31] are used to assess the attention control aspect of executive function. Although ECS score is known to correlate with performance in the Stroop task [25], the relationship between executive function performances assessed with these various tasks and ECS score is not fully understood. In general, an advantageous feature of the use of a specified cognitive task, such as the Stroop task, in a well-controlled laboratory setting is the objective assessment of cognitive abilities, compared with questionnaire-type tests that assess the self-rated (subjective) capacity of cognitive abilities. Nonetheless, questionnaire-type tests can assess cognitive abilities exerted in daily life, without the need for familiarizing with experimental apparatus and task procedures. This is particularly important for elderly participants, to avoid confounding factors such as fatigue and/or stress.

In this study, we examined the validity of using the ECS score as an index of executive function capacity by testing its correlation with risky driving tendencies assessed with the DBQ. The results demonstrated that ECS score was negatively correlated with risky driving tendencies, which is consistent with evidence that executive function capacity is associated with vehicle crash risk in elderly drivers [2]–[7]. This suggests that the ECS is capable of assessing an aspect of executive function relating to daily driving behaviors, and is therefore practically suited to the purpose of this study. However, it should be noted that executive functioning is not the only factor contributing to vehicle crash risk. Risky driving in elderly individuals is known to be associated with various perceptual, cognitive, and physical disabilities [1]. In the present study, correlation coefficients between ECS and DBQ scores (*errors* and *lapses*) were statistically significant, but modest, implying that factors not taken into account in our analyses also contribute to risky driving tendencies.

Moreover, despite several lines of evidence suggesting the implications of DBQ scores in crash risks [32]–[34], DBQ scores per se represent *self-reported* risky driving tendencies. Therefore, if participants had no self-awareness of risky incidents they had perpetrated while driving, the DBQ would underestimate their crash risks. This is a common limitation not only of our study but also of previous studies in which the DBQ has been used to assess crash risks (e.g., [35]–[37]).

### VBM analysis

In the present study, VBM analysis revealed a positive correlation between executive function capacity and gray matter volume in the SMA. Previous functional neuroimaging studies have demonstrated that the SMA is activated in situations where inhibition of incorrect responses is required [38]–[42]. Lesion studies have provided further evidence of a functional role of the SMA in inhibitory control [43]–[46]. Moreover, using transcranial brain stimulation, a causal role of the SMA in response inhibition [47], [48] and task switching [49] has been demonstrated. Collectively, these previous studies strongly indicate that the SMA plays a critical role in the selection and execution of appropriate actions, which is consistent with our result that greater executive function capacity is associated with larger SMA volume.

Our results are also consistent with previous VBM studies demonstrating a positive correlation between executive function capacity and gray matter volume in the frontal lobe [16], [17], [20]. Although the specific frontal lobe regions for which correlations were observed have varied, these studies suggest that larger gray matter volume in the frontal lobe can provide greater computational power for enhanced executive functioning. In contrast, Duarte et al. [19] demonstrated a negative correlation between executive function capacity and gray matter volume in the left middle frontal gyrus. They attributed this result to a narrower variation of cognitive ability in participants recruited as healthy elderly individuals, compared with studies demonstrating the converse relationship. That is, having a large proportion of individuals with poor performance contributes to positive correlations between executive function capacity and gray matter volume in the frontal lobe. However, this explanation might be appropriate for no correlation [18], but seems implausible for the reversal of correlation observed in their study. An alternative possible explanation for the apparent contradiction might be that different regions of the frontal lobe contribute differentially to executive function capacity. For perceptual rivalry of ambiguous figures, higher perceptual switch rate is associated with larger posterior superior parietal lobe volume [50] and smaller anterior superior parietal lobe volume [51]. This can be interpreted as functional segregation of the superior parietal lobe, i.e., the posterior regions detecting alternative perceptual interpretations and the anterior regions preserving the current percept [8]. In this context, the left middle frontal gyrus, whose volume is negatively correlated with executive function capacity [19], might be crucial for a complementary cognitive ability against executive function.

In recent structural neuroimaging studies, in addition to VBM analysis, surface-based morphormetry analysis has been performed to further examine factors contributing to cortical volume changes. For instance, Rimol et al. [52] demonstrated that widespread reductions in cortical volumes in schizophrenia are mainly accounted for by cortical thinning. Thus, we also attempted cortical surface area and thickness analysis on the current data (see Text S1 for details). As a result, although we found a consistent positive correlation of ECS scores with cortical surface area in the SMA, no clusters could survive the correction for multiple comparisons in both cortical surface area and thickness analyses. This might imply that computational power for executive functioning is determined by SMA volume regardless of its components (i.e., surface area and thickness).

Since lower executive functioning is associated with higher vehicle crash risk [2]–[7], our study suggests that smaller SMA volume results in lower executive function capacity, which in turn leads to higher vehicle crash risk. However, in our present study, such a direct relationship between risky driving tendencies and regional frontal gray matter volume was less apparent. This is not a surprising result, because vehicle crash risk in elderly drivers is influenced by perceptual, cognitive and physical disabilities [1]. Therefore, risky driving tendencies that are impacted by numerous factors are not necessarily associated with a specific brain region. To identify brain structural variation associated with risky driving tendencies more accurately, the contributions of such tangled factors must be separately determined.

Finally, the implication of our results is that individual differences in regional frontal gray matter volume might underlie the variation in driving tendencies among elderly drivers. Therefore, detailed driving behavior assessments might be able to detect early neurodegenerative changes in the frontal lobe in normal aging adults; however, further detailed investigation is needed.

## Supporting Information

Text S1 Surface-based morphometry analysis.
(PDF)
