# Supplementary material for: Regional Frontal Gray Matter Volume Associated with Executive Function Capacity as a Risk Factor for Vehicle Crashes in Normal Aging Adults
Source: PLoS One. 2012 Sep 19;7(9):e45920. doi: 10.1371/journal.pone.0045920 (PMC3446941; doi:10.1371/journal.pone.0045920)
Supplement: Text S1 — Surface-based morphometry analysis. (PDF) [file pone.0045920.s001.pdf]

# Text S1. Surface-based morphometry analysis

**Methods.** Using the FreeSurfer software package (http://surfer.nmr.mgh.harvard.edu/), a three-dimensional model of the cortical surface area and thickness measurements was created for each individual. Briefly, pial surface and the gray/white matter boundary were reconstructed on the basis of intensities and continuity information from the entire magnetic resonance volume in segmentation and deformation procedures. Cortical surface area and thickness measurements were smoothed with a 12 mm full-width at half-maximum isotropic Gaussian kernel over the extracted surfaces. Following, a multiple regression was performed vertex-wise to examine correlations between cortical surface measurements and executive function capacity assessed by the Effortful Control Scale (ECS) in the frontal lobe. Age and gender were considered as covariates of no interest to regress out their contributions to cortical surface measurements. The statistical criteria were set to $P < 0.005$ at vertex level, with $P < 0.05$ corrected for multiple comparisons using a Monte Carlo simulation (10,000 iterations) at cluster level.

**Results.** In both cortical thickness and surface area analyses, we could not find any significant cluster showing a correlation between cortical surface measurement and ECS score across individuals. However, it might be noteworthy that in the cortical surface area analysis, the largest clusters showing positive correlations with ECS scores were found in the supplementary motor area (BA 6) in both left and right hemispheres (Figure S1). This finding is consistent with the results in the current voxel-based morphometry analysis.

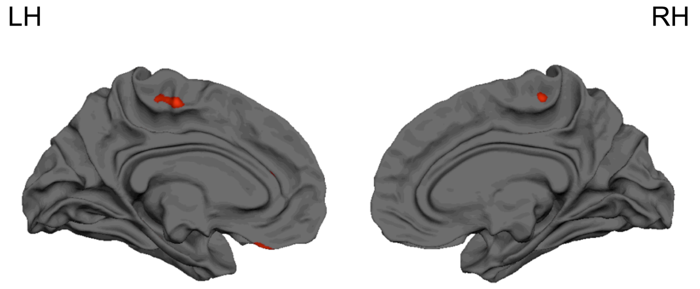


**Figure S1.** Results of cortical surface area analysis. Clusters shown in red indicate that the cortical surface area was positively correlated with executive function capacity assessed by the ECS across individual participants (uncorrected $P < 0.005$). Although none of the clusters did not survive correction for multiple corrections using Monte Carlo simulation ($P < 0.05$, 10,000 iterations), the largest clusters were found within the supplementary motor area (BA 6) in both the left and right hemispheres (LH and RH, respectively). The MNI coordinates of the peak vertices were $x = -6$, $y = -19$, $z = 56$ for LH and $x = -8$, $y = -25$, $z = 55$ for RH, respectively.
